# Hepatitis B and pregnancy: understanding the experiences of care among pregnant women and recent mothers in metropolitan Melbourne

**DOI:** 10.1186/s12889-022-13112-0

**Published:** 2022-04-23

**Authors:** Marvad Ahad, Jack Wallace, Yinzong Xiao, Caroline van Gemert, Gabrielle Bennett, Jonathan Darby, Paul Desmond, Samuel Hall, Jacinta Holmes, Tim Papaluca, Susanne Glasgow, Alexander Thompson, Margaret Hellard, Joseph Doyle, Jessica Howell

**Affiliations:** 1 Burnet Institute, Melbourne, Victoria 3004 Australia; 2 La Trobe University, Bundoora, Victoria 3086 Australia; 3 Centre for Social Research in Health, UNSW Australia, NSW Kensington, 2052 Australia; 4 Department of Gastroenterology, St Vincent’s Hospital, Fitzroy, Victoria 3065 Australia; 5 University of Melbourne, Parkville, Victoria 3010 Australia; 6 School of Public Health and Preventive Medicine, Monash University, Melbourne, Victoria 3004 Australia; 7 Department of Infectious Diseases, The Alfred and Monash University, Melbourne, Victoria 3004 Australia; 8 The Peter Doherty Institute for Infection and Immunity, Melbourne, Victoria 3000 Australia

**Keywords:** Hepatitis B, Pregnancy, Healthcare seeking, Qualitative research

## Abstract

**Background:**

Pregnant women are a priority group for hepatitis B testing. Guideline-based care during antenatal and post-partum periods aims to prevent mother-to-child transmission of hepatitis B virus and lower the risk of liver complications in mothers. This qualitative study explored knowledge of hepatitis B and experiences of hepatitis B related care among pregnant women and mothers.

**Methods:**

Semi-structured interviews were conducted with thirteen women with hepatitis B who were attending antenatal or post-partum hepatitis B care. The interviews were thematically analysed to assess knowledge and understanding of hepatitis B. Participants were recruited from specialist clinics in metropolitan Melbourne between August 2019 and May 2020.

**Results:**

Four major themes were identified from interviews: (1) knowledge and understanding of hepatitis B, (2) treatment pathways, (3) accessing hepatitis B related care, and (4) disclosing status to friends. Most participants displayed an understanding of hepatitis B transmission, including mother to child transmission. The main motivator of post-partum attendance was reassurance gained concerning their child’s health. Sources of hepatitis B information included doctors, online information and family. Participants identified parents and siblings as sources of support and reported an unwillingness to disclose hepatitis B status to friends.

**Conclusions:**

Women attending antenatal or post-partum care reported having overall positive experiences, particularly regarding reassurance of their child’s health, but displayed misconceptions around horizontal transmission. Knowledge gained from these results can contribute to the development of targeted models of care for pregnant women and mothers with young children to ensure their successful linkage to care.

**Supplementary Information:**

The online version contains supplementary material available at 10.1186/s12889-022-13112-0.

## Introduction

An estimated 257 million people globally are chronically infected with hepatitis B virus (HBV) and the most common route of acquisition is from mother to child during birth [1]. Without regular monitoring and timely treatment, people with chronic hepatitis B are at risk of death from liver cirrhosis and liver cancer [2]. In Australia, an estimated 1% of the population are living with hepatitis B, the majority of whom were born overseas in hepatitis B endemic countries (61%), most commonly in North-East Asia (21%) or South-East Asia (17%), where an estimated 30% to 50% have acquired infection perinatally [3, 4]. Among women giving birth in Australia, hepatitis B prevalence varies widely by country of birth between 0.2% and 11%, with a significantly higher prevalence among women born overseas [5].

Vertical transmission, or mother to child transmission (MTCT), of HBV occurs via exposure to blood perinatally and is a key risk factor in hepatitis B infection globally [1, 6, 7]. Rates of MTCT need to be reduced if elimination targets are to be met [1]. As a response to targets set to increase diagnosis and treatment of viral hepatitis, priority populations in Australia were identified in the Third National Hepatitis B Strategy (2018–2022), which included pregnant women alongside people from culturally and linguistically diverse communities [8]. Addressing the barriers to hepatitis B care that Australia faces is critical for eliminating gaps in the cascade of care, ensuring that diagnosis rate goals are met, and that all those diagnosed receive appropriate treatment and management.

To address this, Australia has implemented routine screening of pregnant women for active hepatitis B infection and universal infant vaccination, which resulted in a decrease in newly acquired HBV cases [9–11]. However, gaps still exist in the cascade of hepatitis B care, including the linkage to care of women with hepatitis B during the postpartum period [12, 13] The cascade of care for mothers includes initial screening in pregnancy, referral in the third trimester and post-partum follow up for mothers. While routine screening of pregnant women for hepatitis B infection represents an ideal opportunity to link women with hepatitis B into specialist care, there is an observed loss to follow up in the post-partum period [14].

Barriers to accessing specialist care and being maintained in care have been investigated for key population groups, including people from culturally and linguistically diverse communities in Australia**.** However, there is limited research on the potential barriers to specialist care for pregnant women and recent mothers as a key population. Within this population, there are two care needs that must be considered; firstly, the short-term care needs of the infant and, secondly, the mother’s long-term disease management needs.

An understanding of the experiences of women currently linked into hepatitis B related care could help to inform medical models of care for mothers in order to improve continued engagement in hepatitis B care. An exploratory qualitative design was employed with the aims to, firstly, explore women’s knowledge of hepatitis B and, secondly, explore their experiences in accessing hepatitis B related care during pregnancy and in the post-partum period.

## Methods

This study employed qualitative semi-structured interviews (Additional file 1: Appendix A) to explore women’s experiences in accessing hepatitis B related care during pregnancy and the post-partum period. The study used the consolidated criteria for reporting qualitative research (COREQ) in its development, implementation and reporting (Additional file 1: Appendix B) [15].

### Participants and recruitment

Women diagnosed with hepatitis B over the age of 18 years who were pregnant or had given birth within the preceding 18 months were recruited from specialist liver clinics in two tertiary hospitals in Melbourne, Australia. Recruitment was from August 2019 to May 2020. Potential participants were identified from medical records at specialist clinics and the interviewer then contacted the attending doctor to invite patients to participate in the study. Refusal to participate was not recorded. Face to face interviews held prior to March 2020 were conducted at the service that participants attended, either before or after their specialist consultation. One participant was recruited from the infectious diseases clinic and twelve from the specialist liver clinics. From March 2020 onwards, all interviews were conducted by telephone due to the COVID-19 pandemic that resulted in all outpatient hospital clinic appointments being conducted via telehealth.

Thirteen women aged 24–35 years were interviewed, three of whom were pregnant at the time of interview and ten had given birth in the last 18 months from time of recruitment. Repeat interviews were not performed. Twelve interviews were conducted in English, and one with an accredited Burmese phone interpreter. Participants 1 through 8 were interviewed at the relevant health service face to face, while participants 9 through 13 were interviewed via phone due to COVID-19 restrictions. Table 1 includes characteristics of participants, including cultural background as stated by participants.

**Table 1:** Characteristics of participants, including age, weeks pregnant (where applicable), and cultural background as stated by participants

Participant number	Age	Pregnant at time of interviewing	Self-reported cultural background/country of origin
01	31	Yes (33 weeks)	Chinese
02	24	No	Myanmar
03	34	Yes (20 weeks)	Chinese
04	33	No	Hong Kong
05	33	No	Vietnamese
06	25	Yes (32 weeks)	Vietnamese
07	32	No	Indonesian
08	33	No	Not stated
09	31	No	Fijian Tongan/New Zealand
10	32	No	Nigerian
11	32	No	Chinese
12	32	No	Indian
13	35	No	Malaysian

### Data collection

Demographic information was collected, including maternal age, weeks’ gestation or age of child, contact number, and residential address for distribution of participant information and consent form via mail. Semi-structured interviews were used to collect data using interview prompts based on existing literature and review by co-investigators (Additional file 1: Appendix A). The interview prompts were related to hepatitis B knowledge (including treatment and transmission), sources of information, and previous and current experiences of hepatitis B related health care. The digitally recorded interviews were conducted in a private room at the health service or over the phone. Semi-structured interviews ranged between 13 and 35 min in length, with a mean length of 17 min. Interviews were audio recorded by Dictaphone and field notes were made both during and after interviews by the interviewing researcher.

Data collection stopped after data saturation. Verbal and written consent was provided prior to the interview, after participants were provided with a participant information and consent form, with an option to withdraw their consent at any time.

### Data analysis

Audio files were transcribed by the interviewer and the resulting transcripts were de-identified. Data from interviews were analysed using Braun and Clarke’s criteria for thematic analysis to ensure that data collection and analysis was conducted comprehensively [16, 17]. Data analysis was conducted by one researcher, the interviewing researcher, with guidance from senior researchers. Coding of transcripts began after the first interview and continued as subsequent interviews were conducted. Codes were derived from the data inductively, without the use of a pre-existing coding framework using NVivo12 software (QSR International Pty Ltd. Version 12, 2018). The data was coded inclusively; text surrounding the code of interest was included for context. The data was coded for patterns, frequency, sequence and causation. In the following analytic phase, codes were sorted into potential themes based on their relationship to the research question and to each other. Visual representations were used to form working themes. These themes were then refined, a detailed analysis on each theme was conducted, and themes presented.

### Ethics

This project received ethics approval from the Alfred Health Research Ethics Committee (HREC 149/19).

## Results

### Major themes

Four major themes were inductively identified from participant interviews: (1) knowledge and understanding of hepatitis B, (2) treatment pathways, (3) accessing hepatitis B related care, and (4) disclosing status to friends.

#### Theme 1: Knowledge and understanding of hepatitis B

The majority of participants reported knowing how transmission of hepatitis B occurred. Most participants identified contact with blood as a means of transmission, while a few were unsure whether transmission could occur by contact with blood. Most of the interviewed women were aware of mother to child transmission, with some being aware of mother to child transmission before their pregnancy. One participant expressed that, while she did not know anything at all about hepatitis B, the one thing she wanted to know was how she came to acquire it.

Some participants expressed confusion over whether hepatitis B transmission could occur through sharing of food or cutlery, with one participant expressing confidence in her knowledge of this. A few women noted that while they had previously thought transmission could occur through sharing of food or cutlery, accurate information about transmission had been provided by her specialist during pregnancy. Provision of this information made a substantial change in one woman’s perception of herself:

*Before knowing that [hepatitis B could not be transmitted through sharing food] I just felt very diseased and very gross. I didn’t want to share anything with anyone because I didn’t want to give it to anyone, but after knowing it makes you more at ease.*

*(Participant 6)*

Most of the interviewed women reported searching for hepatitis B information online. Wanting to know more about hepatitis B than what was explained by doctors was the main motivator for online searches. However, women expressed uncertainty over the credibility of information they found online and reported asking doctors for confirmation or advice. As is explained by a woman who was unsure if transmission could occur via sharing of food:

*I tried to see if it could transfer from people to people by sharing drinks and food, but I couldn’t find that information… because I didn’t have the right person to talk to, I was still fifty-fifty percent until I saw the doctor here.*

*(Participant 5)*

In contrast, some women claimed that searching for information online was unnecessary as they felt they had received enough information from doctors, while others avoided searching for additional information so as not to increase their concern.

Family was also cited as a source of new information and support, with women saying that their parents provided support even if they did not significantly know about hepatitis B. All women reported being comfortable in speaking to their close family, which they defined as partners, parents, and siblings about hepatitis B. For those who had hepatitis B during their childhood or adolescence, discussion about family support included the role of parents and their experiences earlier in life.

*My dad had this disease and according to my childhood in my case, we should drink lots of water … and we should eat healthy food.*

*(Participant 12)*

Some women were concerned about having children, and those with sisters who were also living with hepatitis B and who had children, reported their sisters’ experiences during pregnancy as valuable particularly in relation to transmission.

*When we were little, I was thinking “If I have a baby, the baby will carry it on as well.” And at that time, we were young, so I was thinking, “I will never have kids”… But until a few years ago when my sister had a baby and in the hospital in Vietnam… so it gave me hope as well that I want to have a baby.”*

*(Participant 5)*

#### Theme 2: Treatment pathways

The majority of women interviewed said they knew that hepatitis B treatment was available. All participants expressed wanting to know more about treatment when asked. When discussing treatment, participants often included explanations of lowering viral load or viral levels, which in most cases included mention of a hepatitis B “carrier”.

*They [the doctors] said I’m just a carrier in their words—so it’s just a little bit infection it’s not like I literally got it.*

*(Participant 1)*

During discussion of general knowledge of hepatitis B, women expressed uncertainty over their word choice, stating they were unsure if the terms used were ‘correct’ or ‘professional’. Others used words typically found in a biomedical context, particularly when discussing transmission and treatment, such as ‘viral load’, ‘asymptomatic’, ‘natural antibodies’, and ‘antigens’. Participants’ word choice when sharing their hepatitis B knowledge may be a reflection of their main sources of information, in this case, doctors.

Issues related to general health status were raised by participants during discussion of treatment. Participants reported exercise, a healthy diet, and lowering alcohol consumption as key health promoting interventions suggested by doctors, both overseas and in Australia. Discussion of alcohol consumption was linked to concerns of the long-term risk of liver cancer, as explained by one woman:

*When I was young, I was drinking a lot. … But later on, when I was thinking, “Well I can’t keep going like this because one day if it turns out [as] cancer”.*

*(Participant 5)*

In some cases, women reported hearing of potential benefits of herbal medicines from their family, while most claimed to be unsure of their efficacy. One woman recounted her brother’s use of turmeric as treatment for hepatitis B:

*One of my brothers said, because he is a pharmacist, he told us that the other brother has hepatitis B [and] decided not to go through the medical treatment. … my mum gave like turmeric every day and it was really really helpful and he’s fine now.*

*(Participant 7)*

#### Theme 3: Accessing hepatitis B care

This third theme concerned women’s experiences of accessing hepatitis B related care during and after their pregnancies. In interviews, women identified specialists as their main source of hepatitis B related information and recounted their experiences of accessing care throughout their life, including before, during and after pregnancy. Their overall experiences with specialists were positive, with women saying their doctors were important sources of information and their pregnancy provided them with the opportunity to learn more about hepatitis B.

*Actually, I am very happy to go through this because the first thing is, if I was not pregnant, I wouldn’t remember [about hepatitis B] … Probably a lot of people have a problem, but they don’t know.*

*(Participant 1)*

Even though women were attending consultations for their own care, the main concern during pregnancy for participants was the health of their child; most expressed worries about transmitting hepatitis B to their child. Women disclosed that they were satisfied to hear from specialists that transmission could be prevented. Participants also reported that receiving clear and useful information from doctors helped to reduce their worries about their child’s health. One woman explained her initial reluctance to start antiviral therapy:

*… at first, I was like hesitant—reluctant—to start the medication because I thought it might affect the baby. But after they explained everything to me, they convinced me to start the medication…*

*(Participant 8)*

Women also recounted positive experiences during the post-partum period at the liver clinics, except for two cases, where participants explained not being provided with the result of their child’s blood test which was not linked to the institution to which they were attending for their own care. However, follow up of children was still mentioned by participants when discussing their own experiences. The reassurance gained from specialist sessions concerning their child’s health was described as the main motivator for attendance by women.

The importance of continuing to attend appointments during pregnancy was raised during discussion. Participants mentioned that doctors would encourage attendance and mention the benefits of future monitoring for mothers with hepatitis B beyond their pregnancy.

*I was very looked after and if there were any concerns, they made sure that I was monitored and* [they were] *really friendly, very positive.*

*(Participant 9)*

Factors presented as difficulties for attending appointments included distance, parking, not feeling like attending, and for one participant who due to her visa type needed to pay for the cost of consultations. Women placed emphasis on the friendliness of staff and the atmosphere of the waiting areas at the service location they attended. This is exemplified by a participant who cited a reason for not attending her previous clinic:

*Parking was a pain and the place just looked really scary I didn’t like going there very much… ’cause you’re like in a room full of people that you know also have different things as well—and hospitals just look scary.*

*(Participant 6)*

Approximately half of the interviews were conducted by telephone due to the limiting of face to face health service provision as a result of COVID-19 restrictions. Several of the women rescheduled appointments given the disruptions due to the pandemic and preferred using telehealth as it was more flexible. Only two participants expressed preferring attending in person.

*With COVID happening, my last appointment got moved to a telephone appointment, which was so much better. […] it was really good just not having to go the extra distance because I live 45 minutes out of the city at the moment and distance—so having to save me doing that drive with a child is awesome.*

*(Participant 9)*

#### Theme 4: Disclosing status to friends

All women interviewed said they would not usually disclose their hepatitis B status to friends as they feared social exclusion as a result of misconceptions about transmission. Another reason provided was that an individual’s health was a private concern which is not discussed between friends.

*I don’t normally tell my friends that I am a hep B carrier because I don’t know how they would think of me […] they might not know much about hepatitis B like me, so they may avoid being close to me…*

*(Participant 4)*

While friends living with hepatitis B were sources of information for some women, others said they kept sharing of information to a minimum or they preferred not to talk about it at all. Some were comfortable in discussing hepatitis B with their friends in general without disclosing their own status. Participants expressed that they felt responsible for protecting others, which was linked to the misconception that HBV is transmitted by sharing of food. This is explained by one woman who, despite now knowing that transmission cannot occur by sharing of food, continued to feel this responsibility. She describes the mental burden of worrying about potentially infecting others:

*I believed that if we shared drinks or shared foods we may catch it as well. I didn’t want to affect my friends… I didn’t want them to catch whatever I got. […] I’m just afraid with people, knowing that I’ve got something that can’t be treated or no treatment—it’s like a kind of disability thinking.*

*(Participant 5)*

The connotations of the word ‘hepatitis’ were said by some to include a relationship between HIV and hepatitis B, which made them question what their friends would think if they were to share this information with them. The possibility that friends may not understand how hepatitis B was transmitted also made women hesitant to tell their friends.

*Some people may think that “Oh hep B is like HIV!”, you know? “You can get it so easily”—or some people think that if you eat together, they may get it as well.*

*(Participant 4)*

## Discussion

Pregnancy provides a unique opportunity to engage and understand the experiences of women with chronic hepatitis B infection. However, until this work very little research has been undertaken in this area. In this study of firsthand recent experiences of pregnancy, four themes were identified from data collected from women attending hepatitis care, including: (1) knowledge and understanding of hepatitis B, (2) treatment pathways, (3) accessing hepatitis B related care, and (4) disclosing status to friends. Importantly, participants displayed a basic understanding of hepatitis B as a transmissible virus, however, there were certain areas of uncertainty despite their engagement in care. A main driver of attendance was to prevent harm to their baby; and most participants reported positive experiences of care. Nevertheless, our findings suggest that specialist clinics could provide better education, support, and counselling to this group.

Participants displayed a basic understanding of hepatitis B as a transmissible virus, however they still expressed worries regarding horizontal transmission. Misconceptions of transmission through the sharing of food have been identified in the literature among the wider population of people with chronic hepatitis B [18–24]. While participants in this study understood that transmission could not occur by sharing of food, their knowledge did not override their anxiety of transmitting it to others. Most participants were aware of MTCT of hepatitis B and knew that transmission to infants could be prevented. Women expressed concerns regarding potential transmission of hepatitis B to their child both before and during their pregnancy, which was alleviated by reassurance received from specialists while attending the clinic, with particular mention of child immunisation.

The importance of ongoing monitoring and maintaining a healthy lifestyle were discussed in conversations about treatment, as presented in the second theme. Participants reported searching about hepatitis B online when seeking information beyond what was provided by specialists, supporting findings from international quantitative studies on hepatitis B knowledge of mothers where healthcare professionals and the internet were identified as the main sources of hepatitis B information [25–27]. However, the data gathered showed that women prefer hearing from doctors, as they could not be confident of the validity of online information. This opens an opportunity for the promotion of information from a reputable community source that women could rely on.

The third theme derived from the data collected related to the accessing of hepatitis B care during pregnancy and after birth. Participants related positive experiences at their respective clinics, placing an emphasis on the health of their child. Studies in the USA and the UK have identified HBV follow up with mothers after birth to be suboptimal and a lack of a formal referral mechanism was observed [28–30]. In Australia, follow-ups of mothers post-delivery was found to be either lacking or not well documented [6, 31]. Ten of the thirteen participants interviewed in this study were attending appointments after birth at the time of interviewing. In two cases, participants recounted experiencing difficulties in the follow-up process regarding their child. The data shows that women brought up discussion of their child’s health when discussing both their own hepatitis B related care. This highlights a continued emphasis on the child’s health as the main motivator for post-partum appointment attendance, reflecting an underlying area of concern from mothers. Currently, the two care pathways for mother and child for hepatitis B related care are separate. This leads to the question of how to best retain mothers in care and whether the development of a model of care targeted to mothers would lead to increased engagement in specialist care for women during the post-partum period.

The fourth theme dealt with disclosure of hepatitis B status to friends. Stigma and marginalisation of people with hepatitis B is widely reported, which usually occurs after a person discloses their infection [32]. All participants reported they would not typically disclose their hepatitis B status to their friends given their concern over the effect on existing relationships, including the risk of social exclusion, which has been found in other studies [18, 33]. Immediate family members were noted by several participants as a source of information and support and highlights emerging literature on the supportive role of families in blood borne viruses [34].

### Strengths, limitations, and future directions

A strength of this study was that it focussed on a key population on which little research has been undertaken previously. Qualitative inquiry in this area allowed for understanding the experiences of care during pregnancy and after birth from the perspectives of women themselves. Additionally, an inductive approach to thematic analysis allowed for themes to be derived directly from the first-hand experiences of the women who participated in the study,

Limitations of this study include the possibility of recruitment bias given that recruited participants were already linked into care and actively attending specialist clinic appointments for their own hepatitis B care. Actual barriers to attendance may be different from the perceived barriers shared by women who were linked into care and attending appointments. Additionally, while confidential, interviewed participants may have been concerned that results may impact their relationship with the service provider. Twelve of thirteen interviews were conducted in conversational English, and one by phone interpreter. As previous studies have identified limited English proficiency as one of the barriers to receiving adequate HBV education during pregnancy, future qualitative studies should include non-English speaking participants [6].

Additionally, future qualitative studies should engage pregnant women and recent mothers who are not currently linked into care. The data gained from this study could also be used for the development of quantitative studies concerning hepatitis B knowledge of pregnant women or assessment of engagement in care.

## Conclusions

Women living with hepatitis B had positive experiences of antenatal and post-partum hepatitis B specialist care, especially regarding reassurance of their baby’s health, their own health and the importance of ongoing monitoring. Findings from this study suggest that specialist clinics could support better education and support for pregnant women and recent mothers with hepatitis B. Diagnosis and engagement in care is currently suboptimal in Australia and is a challenge in the field. As a poorly researched area, the themes identified in this explorative qualitative study show further avenues that need to be explored in order to improve hepatitis B care services for mothers post-partum. Further investigation could inform the design of specialist medical services to improve their accessibility and ensure successful linkage to care of mothers.

## Supplementary Information

**Additional file 1: Appendix A.** Semi-structured interview guide. **Appendix B.** COREQ (COnsolidated criteria for REporting Qualitative research) Checklist.

## Data Availability

The datasets generated during and analysed during the current study are not publicly available due to limitations of ethical approval involving the participant data and anonymity, but are available from the corresponding author on reasonable request.

## Abbreviations

HBV: Hepatitis B virus
MTCT: Mother to child transmission
