# Supplementary material for: Hepatitis B and pregnancy: understanding the experiences of care among pregnant women and recent mothers in metropolitan Melbourne
Source: BMC Public Health. 2022 Apr 23;22:817. doi: 10.1186/s12889-022-13112-0 (PMC9034770; doi:10.1186/s12889-022-13112-0)
Supplement: Supplementary file 1 — Additional file 1: Appendix A. Semi-structured interview guide. Appendix B. COREQ (COnsolidated criteria for REporting Qualitative research) Checklist. [file 12889_2022_13112_MOESM1_ESM.docx]

**Appendix A: Semi-structured interview guide**

**Demographics**

We might start first with some questions about you.

- How old are you?
- What is your background?
- How many weeks pregnant?/When did you give birth?

**General hepatitis B knowledge**

First let’s talk about hepatitis B in general.

- Could you share some of the things you know about hepatitis B?

*Probes*

What do you know about the way its transmitted?

**Sources of hepatitis B information**

- Where have you heard about hepatitis B?
  - Who have you talked to about hepatitis B?
  - Why have you chosen to speak to them about hepatitis B?

*Probes*

From who have you heard about hepatitis B?

**Previous experiences with hepatitis B/ sources of hepatitis B information**

- Do you know anyone who has hepatitis B?
  - What have you learnt from them?
  - What have they told you about hepatitis B?

*Probes*

Do any of your family and friends have hepatitis B?

**Sharing information/ sources of hepatitis B information**

- Who have you told about having hepatitis B?
  - Why did you choose to tell them?
  - What have they told you about hepatitis B?

**Knowledge of hepatitis B treatment**

- Could you tell me what you know about hepatitis B treatment?

*Probes*

Do you know what the reason for treatment is?

**Knowledge of hepatitis B transmission**

Let’s now talk about transmission of hepatitis B.

- What do you know about hepatitis B transmission? Could you explain it?
  - How can it be stopped?
  - What do you know of hepatitis B transmission between partners?
  - Do you know how it can be avoided?

**Current experiences**

- Are you happy with the information you got from your doctor?
  - Are there any things that could have been better?

**Appendix B: COREQ (Consolidated criteria for Reporting Qualitative research) Checklist**

**Domain 1: Research team and reflexivity**

*Personal characteristics*

Which author/s conducted the interview or focus group? MA

What were the researcher’s credentials? BBmed, Bsc(Hons)

What was their occupation at the time of the study? Student (2019), Research Assistant (2020)

Was the researcher male or female? Female

What experience or training did the researcher have? Experience conducting qualitative interviews and thematic analysis of qualitative data.

*Relationship with participants*

Was a relationship established prior to study commencement? No

What did the participants know about the researcher? Occupation and reasons for interviewing.

What characteristics were reported about the interviewer/facilitator? Educational background, objectives and aims of research topic.

**Domain 2: Study design**

*Theoretical framework*

What methodological orientation was stated to underpin the study? Phenomenology.

*Participant selection*

How were participants selected? Purposive sampling.

How were participants approached? Potential participants were told of study by their doctors and then introduced to the interviewer who took written consent.

How many participants were in the study? Thirteen.

How many people refused to participate or dropped out? Reasons? Refusal to participate was not recorded.

*Setting*

Where was the data collected? For in person interviews, in a separate and private room at the service location participants were attending. The remainder were conducted via telephone call.

Was anyone else present besides the participants and researchers? No.

What are the important characteristics of the sample? Clinic attendance, and pregnancy/recent birth.

*Data collection*

Were questions, prompts, guides provided by the authors? Was it pilot tested? An interview guide was developed by collaboration with senior researchers.

Were repeat interviews carried out? If yes, how many? Repeat interviews were not carried out.

Did the research use audio or visual recording to collect the data? Yes, a Dictaphone was used to audio record interviews.

Were field notes made during and/or after the interview or focus group? Yes, field notes were made both during and after interviews.

What was the duration of the interviews or focus group? Interviews lasted between 13 and 35 minutes.

Was data saturation discussed? Yes data saturation was discussed with senior researchers.

Were transcripts returned to participants for comment and/or correction? No.

**Domain 3: Analysis and findings**

*Data analysis*

How many data coders coded the data? One.

Did authors provide a description of the coding tree? No.

Were themes identified in advance or derived from the data? Themes were derived from the data.

What software, if applicable, was used to manage the data? NVivo12 (QSR International Pty Ltd. Version 12, 2018.)

Did participants provide feedback on the findings? No.

*Reporting*

Were participant quotations presented to illustrate the themes/findings? Was each quotation identified? Yes participant quotations were presented with corresponding participant number.

Was there consistency between the data presented and the findings? Yes.

Were major themes clearly presented in the findings? Yes.

Is there a description of diverse cases or discussion of minor themes? Yes.
